# Melanocortin-3 receptors are involved in adaptation to restricted feeding

**DOI:** 10.1111/j.1601-183X.2012.00766.x

**Published:** 2012-04

**Authors:** K Begriche, O J Marston, J Rossi, L K Burke, P McDonald, L K Heisler, A A Butler

**Affiliations:** ‡ Department of Metabolism and Aging, The Scripps Research InstituteJupiter, FL, USA; ‡ Department of Pharmacology, University of CambridgeCambridge, United Kingdom; § Department of Molecular Therapeutics and Translational Research Institute, The Scripps Research InstituteJupiter, FL, USA

**Keywords:** Appetite, dorsomedial hypothalamus, hypothalamus, melanocortin, melanocortin-3 receptor, nutrient sensors

## Abstract

The central nervous melanocortin system forms a neural network that maintains energy homeostasis. Actions involving neural melanocortin-3 receptors (MC3Rs) regulate the expression rhythms in ingestive behaviors and metabolism anticipating nutrient intake. Here, we characterized the response of *Mc3r* knockout (*Mc3r*^−/−^) and wild type (WT) mice to a restricted feeding (RF) schedule where food access was limited to a 4-h period mid light cycle using a mechanical barrier. *Mc3r*^−/−^ mice adapted poorly to the food restriction schedule. Anticipatory activity and the initial bout of intense feeding activity associated with granting food access were attenuated in *Mc3r*^−/−^ mice, resulting in increased weight loss relative to controls. To investigate whether activity in specific hypothalamic nuclei contribute to the *Mc3r*^−/−^ phenotype observed, we assessed hypothalamic FOS-immunoreactivity (FOS-IR) associated with food restriction. Food access markedly increased FOS-IR in the dorsomedial hypothalamus (DMH), but not in the suprachiasmatic or ventromedial hypothalamic nuclei (SCN and VMN, respectively) compared to *ad libitum* fed mice. *Mc3r*^−/−^ mice displayed a significant reduction in FOS-IR in the DMH during feeding. Analysis of MC3R signaling *in vitro* indicated dose-dependent stimulation of the extracellular signal-regulated kinase (ERK) pathway by the MC3R agonist d-Trp(8)-*γ*MSH. Treatment of WT mice with d-Trp(8)-*γ*MSH administered intracerebroventricularly increased the number of pERK neurons 1.7-fold in the DMH. These observations provide further support for the involvement of the MC3Rs in regulating adaptation to food restriction. Moreover, MC3Rs may modulate the activity of neurons in the DMH, a region previously linked to the expression of the anticipatory response to RF.

The central nervous melanocortin system maintains energy homeostasis through actions mediated by two melanocortin receptors (MC3R and MC4R) (Mountjoy 2011). While the functions of MC4Rs with respect to energy homeostasis have been intensively investigated, less is known about the specific roles of MC3Rs (Renquist *et al.* 2011). However, MC3Rs are clearly required for appropriate energy homeostasis. Deletion of the mouse *Mc3r* gene causes an obesity syndrome (Butler *et al.* 2000; Chen *et al.* 2000), and some reports indicate that *MC3R* polymorphisms that impact on signaling may be associated with increased risk of childhood obesity (Feng *et al.* 2005; Mencarelli *et al.* 2011; Savastano *et al.* 2009; Zegers *et al.* 2011). Experiments examining the response of melanocortin receptor knockout mice to non-selective melanocortin receptor agonists indicate that functional MC3Rs are not necessary for agonist-induced effects on energy expenditure (EE), autonomic function, satiety and weight loss (Begriche *et al.* 2009; Kumar *et al.* 2009). Thus, the functional roles of MC3Rs in the regulation of energy homeostasis by the endogenous melanocortin ligands remain to be clarified.

Recent data suggest that MC3Rs modulate the expression of rhythms that anticipate nutrient availability (Begriche *et al.* 2011a,b; Sutton *et al.* 2008, 2010). Mice subjected to restricted feeding (RF) involving a reduced amount of food administered at 24-h intervals display increased vigilance and locomotory behavior anticipating food presentation, a response also known as food anticipatory activity (FAA). Analysis of the response of MC3R-deficient mice to RF indicates that this receptor may be a point of convergence for inputs into systems regulating rhythms in vigilance and metabolism in response to signals of nutrient consumption.

Several studies have reported that the expression of FAA involves a distributed network of neurons in the DMH, ventromedial nucleus (VMN) and lateral hypothalamus (LHA) (Arble *et al.* 2010). Neurons residing in these areas exhibit rapid regulation in response to food intake, suggesting the involvement of inputs from systems sensing nutrient consumption (Johnstone *et al.* 2006; Singru *et al.* 2007). This response can be inhibited by intracerebroventricular administration of melanocortin receptor antagonist, indicating that melanocortin neurons are at least partially responsible (Singru *et al.* 2007). Arcuate hypothalamic neurons that release the endogenous melanocortin ligands also exhibit rapid regulation by food intake (Johnstone *et al.* 2006; Singru *et al.* 2007). Melanocortin-3receptors are expressed in these areas (Roselli-Rehfuss *et al.* 1993); however, whether they are involved in mediating the effects of feeding-related signals on neural activity is unclear.

## Methods and procedures

### Experimental animals

Experiments using mice were approved by the Scripps Institutional and Animal Care and Use Committee. Wild type (WT) and *Mc3r*^−/−^ mice were obtained from mating male and female *Mc3r*^+/−^ mice in a colony maintained at Scripps Florida and genotyped as described previously (Sutton *et al.* 2008). *Mc3r*^+/−^ breeders backcrossed >10 generations onto the C57BL/6J (B6) background originated from stock provided by Dr Roger Cone and the Oregon Health & Science University (Butler *et al.* 2000). All experiments used male mice aged 2.5–3.5 months that were weaned onto standard rodent chow and maintained on a standard 12-h light:dark cycle (lights on 0700–19:00 h).

### Analysis of behavioral and metabolic adaptation to restricted food access

**Figure 1:**
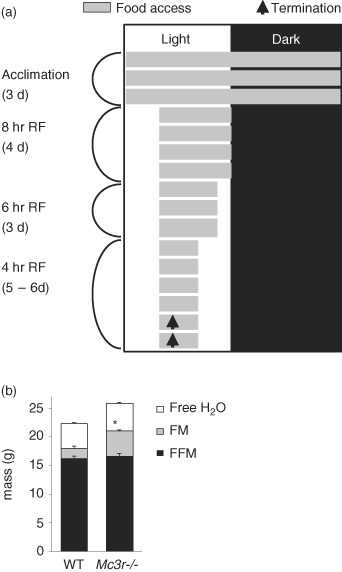
RF protocol and body composition of WT and *Mc3r*^−/−^ mice (a) Schematic representation of the RF protocol. After a 3-day acclimation period, food access (indicated by gray shading) was gradually restricted using a mechanical barrier. Mice were euthanized 90 min after food access on the days indicated by the *arrow*, and brains collected for immunohistochemical analysis. (b) *Mc3r*^−/−^ mice (*n* = 9) had a modest but statistically significant increase in fat mass (FM) relative to WT; water content and FFM were not significantly affected by genotype.

A purified low fat diet, Research Diets 12450B (Research Diets, New Brunswick, NJ, USA), used previously to investigate FAA in *Mc3r*^−/−^ mice (Sutton *et al.* 2008) was employed in these studies. Behavioral and metabolic adaptation to the RF protocol was assessed using a comprehensive laboratory animal monitoring system (CLAMS; Columbus Instruments, Columbus, OH, USA) in a temperature-controlled environment (24°C) (Begriche *et al.* 2011a; Sutton *et al.* 2008). The system is configured to allow simultaneous measurement of food intake, EE and movements in the X- and Z-axis using photocells that monitor infrared beams. The chambers are configured with a center feeder for measuring feeding behavior (meal time, duration and size); access to food was controlled using a pneumatic-driven shield. Mice were allowed unrestricted access to food during a 3-day acclimation period; food access was then gradually restricted using a mechanical barrier to a 4-h period (1100–1500 h) mid light cycle (Fig. 1a). On the final day, mice were euthanized 90 min after food access and brain tissue was prepared for immunohistochemical analysis. Due to the numbers involved, tissue collection was performed over a 2-day period.

For movements in the X-axis, data is analyzed as X(Amb) and X(Tot). X(Amb) refers to the number of ambulatory X-axis infrared beam breaks (i.e. the initiation of movement). For X(amb), repeated interruptions of the same infrared beam do not incur additional ambulatory counts. X(Tot) refers to the total number of infrared beam breaks. Generally, X(Tot) and X(Amb) data were in agreement. To assess FAA, recordings of continuous movements (per minute) in the 4 h preceding and 1.5 h after food access were analyzed on the day of euthanasia. To account for the reduced activity of *Mc3r*^−/−^ mice, for the assessment of FAA, all data were normalized to the total 24-h activity of each mouse.

Energy expenditure was calculated from recordings of oxygen consumption (VO_2_) and the respiratory exchange ratio (RER, the ratio of CO_2_ exhaled to oxygen consumed) applied to the following equation: EE = (3.815 + 1.232 × RER) × VO_2_. Non-resting EE (NREE) and resting EE (REE) were calculated as described previously (Sutton *et al.* 2006). Briefly, REE was calculated using the lowest 10% of recordings assumed to represent periods of minimal activity. Non-resting EE was then calculated by subtracting REE from total 24-h EE. The thermic effect of food was calculated by subtracting the averaged EE data obtained during the period of food access from the average obtained 4 h before food access. The area under the curve for the increase in RER during food access was calculated using the 1-h bin data. Body composition was assessed by measuring fat mass and fat-free mass (FFM) using nuclear magnetic resonance (Mice Minispec NMR Analyzer; Bruker Optics Inc., Billerica, MA, USA).

### Cannulation and intracerebroventricular injections

Briefly, mice were anesthetized with ketamine HCl/xylazine HCl and secured in a stereotaxic apparatus. A guide cannula (26GA; Plastics One Inc., Roanoke, VA, USA) was implanted at the following coordinates: 0.34 mm posterior to bregma, 1 mm lateral to midline and 2.3 mm below the surface of the skull and fixed in place using Loctite 454 acrylate adhesive (Plastics One Inc.). Mice were housed separately for at least 1 week to recover from surgery. At the end of the recovery period, the position of the cannula was verified by intracerebroventricular administration of angiotensin II (10 ng; Sigma-Aldrich, St Louis, MO, USA) in 1 µl Acsf (Tocris Bioscience, Minneapolis, MA, USA). Only those mice that drank immediately after injection were used in experiments. At the end of the experiments, additional confirmation of correct cannula placement was carried out using histological techniques. Intracerebroventricular infusions were performed using a Hamilton syringe attached to PE 50 tubing and an internal guide cannula extending beyond the guide cannula into the lateral ventricle. In the morning of the experiment, mice received intracerebroventricular injections of either Acsf (1 µl) or d-Trp(8)-*γ*MSH (1 nmol; Phoenix Pharmaceuticals Inc., Burlingame, CA, USA) suspended in 1 µl Acsf and 15 min later they were euthanized and brain tissue was collected for immunohistochemical analysis as described below.

### Immunohistochemistry

Male B6 mice purchased from the Jackson Laboratory (Bar Harbor, ME, USA) were either provided with continuous access to food or restricted access in the home cage. Mice with restricted access were gradually acclimated to 4 h of food per day during the light cycle. On the final day, mice were euthanized 90 min after food access and brain tissue was prepared for immunohistochemical analysis and compared with *ad libitum* fed mice. Mice were deeply anesthetized with ketamine/xylazine/acepromazine cocktail (100/10/2 mg/kg, ip) and transcardially perfused with saline and 10% neutral-buffered formalin (Sigma-Aldrich). Brains were post-fixed in 10% formalin for 4 h before being cryoprotected in 20% sucrose in phosphate-buffered saline (PBS) (4°C) and cut into 30-µm thick coronal sections using a freezing sliding microtome. Sections were collected in four equal, adjacent series and stored in antifreeze solution (30% ethylene glycol and 20% glycerol in dH_2_O) at −20°C until use. Series were washed (6 × 8 min) in PBS (Sigma-Aldrich), incubated for 20 min in 1.5% hydrogen peroxide solution (Sigma-Aldrich) and then rewashed (6 × 8 min in PBS). Tissue was then incubated for 60 min in a blocking solution of PBS supplemented with 1% bovine serum albumin (BB international, Cardiff, UK) and 0.04% triton-X-100 (Sigma-Aldrich) and incubated overnight in blocking solution containing a rabbit anti-cFOS primary antibody (1:10000; Calbiochem, San Diego, CA, USA) or rabbit anti-Phospho-p44/42 MAPK (Erk1/2) antibody (1:8000; Cell Signaling Technology Inc., Danvers, MA, USA). Tissue was then washed (6 × 8 min in PBS) and incubated for 90 min in blocking solution containing a donkey anti-rabbit secondary antibody (1:1000; Jackson ImmunoResearch Laboratories, West Grove, PA, USA). Subsequently, the tissue was washed (6 × 8 min in PBS) and incubated for 90 min in PBS containing avidin–peroxidase complex (ABC, 1:250, Vector Elite kit; Vector laboratories, Burlingame, CA, USA). Tissue underwent a final series of washes (6 × 8 min in PBS) before immunoperoxidase staining was developed with a nickel-intensified 3,3′-diaminobenzidine tetrahydrochloride kit (Vector Laboratories). The reaction was terminated by rinsing sections with PBS. Tissue was mounted onto superfrost slides and dehydrated through graded alcohols, cleared in xylene and cover-slipped using micromount (Surgipath Medical Industries, Richmond, IL, USA). Stained sections were examined using both brightfield and darkfield microscopy using a Zeiss Axioskop II with attached charge-coupled device camera (Carl Zeiss Microscopy, Hertfordshire, UK). Bregma levels were assigned to the sections using the Mouse Brain in Stereotaxic Co-ordinates (Paxinos and Franklin) and digital images taken using Axiovision 4.3 software (Carl Zeiss Microscopy). Bregma levels analyzed were DMH (bregma levels: −1.58, −1.82 and −2.06 mm), VMN (bregma level: −1.58 mm), suprachiasmatic nucleus (SCN; bregma levels: −0.34, −0.58 and −0.82 mm), arcuate nucleus (ARC; bregma level: −1.58 mm) or paraventricular hypothalamic nuclei (PVN; bregma level: −0.82 mm). Areas to be analyzed were digitally delineated and FOS-immunoreactivity (IR) or phosphorylated extracellular signal-regulated kinase (pERK)-IR nuclei within these areas were counted by a researcher blind to experimental conditions.

### Cell culture and transfection

Consumables for cell culture were purchased from Life Technologies, Grand Island, NY, USA. Chinese hamster ovary (CHO) cells were cultured in F12K medium supplemented with 10% fetal bovine serum (FBS) and 1% penicillin/streptomycin antibiotics. Cells were incubated at 37°C in humidified air containing 5% CO_2_. The day before transfection, cells were plated into 6-well culture dish at a density of 5 × 10^5^ cells/well and allowed to attach overnight. Cells were approximately at 80–90% confluence on the day of transfection using pcDNA3.1 hMC3R encoding the human MC3R with a 3× HA Tag on the N terminus (Missouri S&T cDNA Resource Center, Rolla, MO, USA). Transfection used the lipofectamine 2000 reagent according to the manufacturer's instruction (Invitrogen, Grand Island, NY, USA). Briefly, CHO cells in 2 ml of medium were transfected with 4 µg pcDNA3.1hMC3R in the instructions of Opti-MEM medium (Invitrogen) and 10 µl of lipofectamine. After 24 h, cells were detached from wells with trypsin–ethylenediaminetetraacetic acid (EDTA) (0.25%), collected by centrifugation and suspended in sterile sorting buffer (PBS1X (−Mg^2+^/−Ca^2+^), 1 mm EDTA, 25 mm Hepes, 1% FBS heat inactivated and Dnase II 10 U/ml). Single cells separated into 96 well plates using fluorescence-activated cell sorter (FACS) cells were cultured in a selective medium containing the above medium supplemented with geneticin (600 µg/ml). Geneticin-resistant clones were transferred in a 6-well culture dish and T75 flask for further selection using an anti-HA antibody to confirm hMC3R expression.

### Cell signaling assays

Cellular cAMP generation was measured by using homogenous time-resolved fluorescence (HTRF) technology (cAMP dynamic kit; Cisbio, Bedford, MA, USA). Briefly, cells were seeded at a density of 1000 cells/well in 384-well low volume plate (Greiner Bio-One North America Inc., Monroe, NC, USA) and incubated overnight at 37°C/5% CO_2_. On the day of the assay, cells were stimulated with various concentrations of a specific MC3R agonist d-Trp(8)-*γ*MSH (Phoenix Pharmaceuticals Inc.). d-Trp(8)-*γ*MSH was diluted in assay buffer (OptiMem medium supplemented with 2% FBS, 200 µm isobutylmethylxanthine and 1.5% dimethyl sulfoxide). After 30-min stimulation at room temperature, cells were then incubated with cAMP-d2 conjugate and cryptate conjugate for 1 h at room temperature in dark conditions. Homogenous time-resolved fluorescence signals were read using an EnVision multilabel plate reader (PerkinElmer, Waltham, MA, USA). Phosphorylated ERK1/2 expression was detected using HTRF technology (Cellul’erk kit; Cisbio), a sandwich immunoassay using an anti-pERK1/2 antibody labeled with d2 and an anti-ERK1/2 antibody labeled with Eu^3+^-cryptate. Cells seeded at a density of 1000 cells/well in 384 well plates were incubated overnight at 37°C in 5% CO_2_. After serum starvation overnight, cells were stimulated with various concentrations of d-Trp(8)-*γ*MSH. After 10-min stimulation, cells were lysed and incubated with d2 conjugate and cryptate conjugate for 2 h at room temperature in dark conditions. Homogenous time-resolved fluorescence signals were read using an EnVision multilabel plate reader.

For western blotting, parental and hemagluttinin-tagged (HA)-hMC3R cells seeded at a density of 5 × 10^5^ cells/well in 12 well plates were cultured in F12K medium supplemented with 10% FBS and 1% penicillin/streptomycin. At 90% confluency, cells were serum starved overnight, washed once with PBS and then stimulated with 1 µmd-Trp(8)-*γ*MSH for 5 or 10 min. After stimulation, cells were rinsed twice with PBS to remove residual media; 100 µl of ice cold RIPA buffer was then added. Lysate was scraped and insoluble material removed by centrifugation (14 000 ***g*** for 10 min at 4°C). Protein concentration of the supernatant was determined using the BCA Protein Assay Kit (Pierce Biotechnology, Rockford, IL, USA). To assess pERK expression, 15 µg of protein was resolved using 4–20% sodium dodecyl sulfate-polyacrylamide gels (Bio-Rad, Hercules, CA, USA), transferred onto nitrocellulose membranes and probed overnight at 4°C with a rabbit polyclonal anti-phospho-p42/p44 MAPK antibody (Cell Signaling Technology Inc.). Membranes were probed with anti-rabbit secondary antibodies (Sigma-Aldrich) and the expression was detected by chemiluminescence. Total ERK was measured using stripped membranes incubated with a monoclonal mouse antibody against p42/44 ERK (Cell Signaling Technology Inc.).

### Statistical analysis

All data are presented as mean ± SEM. Effects of genotype were analyzed with student's *t*-test (with Bonferroni's correction, where appropriate), two-way analysis of variance (anova) or repeated measures anova with *post hoc* comparisons, where appropriate, using Microsoft Excel 2007 and/or SPSS v.19 (IBM, Armonk, NY, USA). Strength and significance of associations in movement between time points leading up to food presentation was assessed using regression analysis. Statistical significance was set at *P* < 0.05.

## Results

### Metabolic parameters during ad libitum conditions

**Figure 2:**
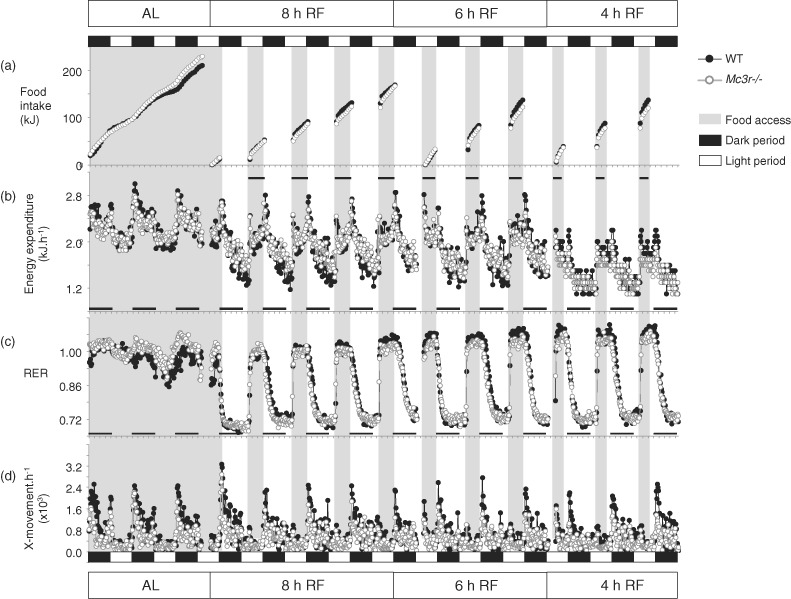
Impact of RF on food intake, EE, RER and activity in *Mc3r*^−/−^and WT mice Data shown are recordings taken at 18-min intervals and averaged by genotype; breaks in the data stream indicate times when the system was dismantled for cleaning. Dark and light periods are indicated by the black and white rectangles situated above the top panel; timing of food access is indicated by gray shading. (a) Cumulative food intake. (b) EE data expressed as kJ/h/mouse. EE declined with RF, food access was associated with increased EE due to the thermic effect of food. (c) RER data. RER declined to 0.7 in the intervals between food access irrespective of genotype, indicating mobilization of fat reserves. An increase in the RER during feeding is consistent with oxidation of the low fat diet. (d) Physical activity was assessed using X-movements.

Male *Mc3r*^−/−^ mice (*n* = 9) and WT littermate controls (*n* = 7) acclimated to housing conditions in the CLAMS for 3 days were subjected to the RF protocol where timing of food access was gradually restricted to a 4-h period mid light cycle (Fig. 1a). The *Mc3r*^−/−^ mice displayed a modest increase in body weight (weight of *Mc3r*^−/−^ mice, 30.6 ± 0.8 g; WT, 27.5 ± 1.0 g; *t*_(14)_ = 2.551, *P* < 0.05) due to increased fat mass (Fig. 1b). *Mc3r*^−/−^ mice exhibited a metabolic profile consistent with previous observations (Butler 2006; Sutton *et al.* 2008). No marked genotypic differences were observed in food intake or expenditure expressed as kJ/h/mouse (Fig. 2a,b). Total EE expressed in kJ/day was not significantly affected by genotype (52.9 ± 0.8 vs. 53.9 ± 1.4 kJ/day for *Mc3r*^−/−^ and WT mice, respectively). Resting EE was also not affected by genotype (44.9 ± 0.6 vs. 43.6 ± 1.3 kJ/day). However, NREE was significantly lower in *Mc3r*^−/−^ mice relative to controls (8.1 ± 0.5 vs. 10.2 ± 0.5 kJ/day; *t*_(14)_ = 3.022, *P* < 0.01). A similar outcome was observed when REE and NREE were expressed per kilograms of FFM (REE in kJ/kgFFM/day for *Mc3r*^−/−^ mice: 2716 ± 36; for WT: 2687 ± 90; NREE in kJ/kgFFM/day for *Mc3r*^−/−^ mice: 488 ± 27; for WT: 630 ± 30; *t*_(14)_ = 3.469, *P* < 0.005). When adjusted for total body weight, a significant difference was observed in both NREE (287 ± 18 vs. 404 ± 19 kJ/kg/day; *t*_(14)_ = 4.533, *P* < 0.001) with a trend for a reduction in REE (1595 ± 34 vs. 1725 ± 60 kJ/kg/day; *t*_(14)_ = 2.001, *P* = 0.065).

*Mc3r*^−/−^ mice also displayed a higher RER, an indicator of the ratio of fat vs. glucose oxidation, during the dark period (1.029 ± 0.003 vs. 0.992 ± 0.010; *t*(7.229) = 3.692, *P* < 0.01). *Mc3r*^−/−^ mice also displayed a reduction in locomotor activity during the dark period (742 ± 57 vs. 1200 ± 151 X-beam breaks/h; *t*(7.725) = 2.838, *P* < 0.05) (Fig. 2c,d), suggesting that the reduction in NREE may be at least partially explained by reduced activity. Respiratory exchange ratio and locomotor activity during the lights-on period were not significantly different between genotypes (RER: 0.980 ± 0.004 vs. 0.963 ± 0.011; movement: 263 ± 24 vs. 309 ± 37 X-beam breaks/h).

### Metabolic parameters during RF conditions

Temporal restriction of food access was associated with reduced cumulative food intake, irrespective of genotype (Fig. 2a). Reduced caloric intake resulted in a decline in EE, particularly during prolonged intervals between meals (Fig. 2b). The RER declined to approximately 0.7 between meals, indicating a shift to fat oxidation when access to food was denied, and rapidly increased when food access was granted (Fig. 2c). The overall rhythm in physical activity appeared normal during RF (Fig. 2d); however, as shown later, RF had significant effects on locomotor activity.

**Figure 3:**
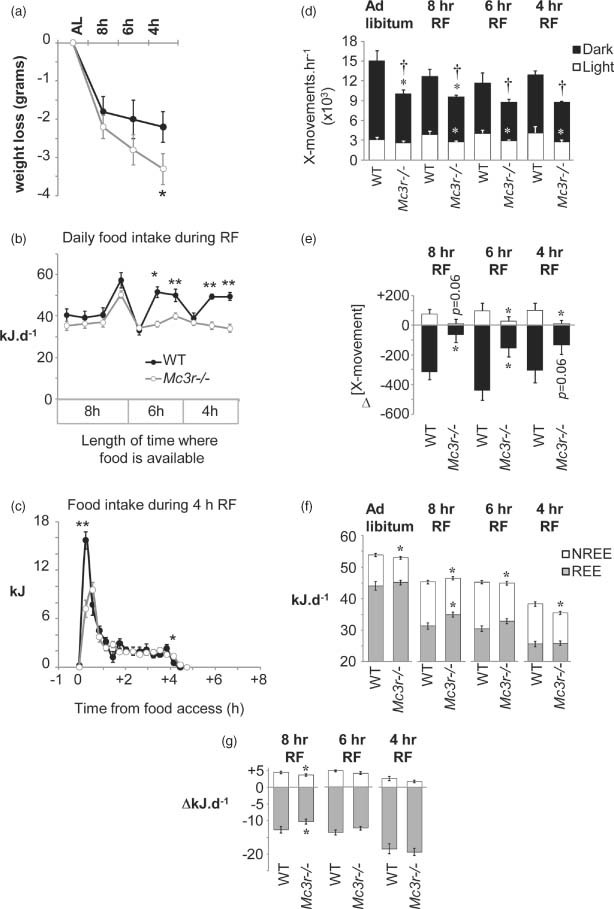
Impact of RF on body weight, food intake, locomotor activity and EE (a) Weight loss associated with RF was similar between genotype when food access was limited to 8 h, but was significantly greater in *Mc3r*^−/−^ mice relative to WT controls with shorter periods of food access. *P < 0.05 vs. WT. (b) Twenty-four-hour food intake during 8, 6 or 4 h of RF. *Mc3r*^−/−^ mice removed significantly less food from the container when food access was limited to 6 or 4 h of RF. *P < 0.05, **P < 0.01 vs. WT. (c) Food intake during 4-h RF shown as 18-min bins; each bin is an average of 2 days of recordings. *Mc3r*^−/−^ mice consumed fewer kilojoules during the first 36 min of food access, with normal intake thereafter. (d) *Mc3r*^−/−^ mice displayed a significant reduction in 24-h activity in the dark period, irrespective of the timing of food access (^†^*P* < 0.05). This was primarily due to a significantly lower level of activity during the dark period, although a reduction in activity during lights-on was also observed during RF (*P < 0.05 when comparing activity between genotype within dark or light period). (e) Net change in locomotor activity in the dark and lights-on periods during RF. The modest increase in activity during the light period observed in WT was attenuated in *Mc3r*^−/−^ mice (*P < 0.05). A decline in activity during the dark period was also significantly attenuated in *Mc3r*^−/−^ mice (*P < 0.05). (f) Analysis of REE and NREE. NREE was lower in *Mc3r*^−/−^ mice, irrespective of feeding conditions (*P < 0.05). REE in *ad libitum* conditions was not significantly affected by genotype; however, during RF REE was higher in *Mc3r*^−/−^ mice when food access was limited to 8 h (*P < 0.05). (g) Net change in NREE and REE during RF. The impact of RF on total EE was due to reduced REE, with NREE appearing to be increased. The impact of restricting food access to 8 h on NREE and REE was significantly attenuated in *Mc3r*^−/−^ mice (*P < 0.05).

The reduction in calorie intake during RF caused weight loss (Fig. 3a) that was most severe during the first 4 days when access to food was limited to 8 h (1.8 ± 0.4 and 2.2 ± 0.4 g for WT and *Mc3r*^−/−^ mice, respectively). Weight loss of WT mice during 6- and 4-h RF was more modest, suggesting adaptation. However, weight loss during 6- and 4-h RF was more pronounced for *Mc3r*^−/−^ mice. Analysis of the impact of RF on weight loss using a one-way anova with repeated measures indicate a significant effect of feeding conditions (*F*_2,28_ = 21.176, *P* < 0.01) and a significant interaction between feeding conditions and genotype (*F*_2,28_ = 3.591, *P* < 0.05). The amount of weight loss observed at the end of the RF protocol was significantly greater in *Mc3r*^−/−^ mice compared to WT (Fig. 3a; *t*_(14)_ = 1.813, *P* < 0.05). The increased weight loss of *Mc3r*^−/−^ mice during RF was at least partially explained by lower food intake (Fig. 3b). Further analysis of food intake of WT mice during the RF period suggested an initial period of gorging followed by a period of more modest feeding behavior with approximately 20 kJ consumed in the first 36 min followed by an intake of 5–6 kJ/h thereafter (Fig. 3b,c). *Mc3r*^−/−^ mice displayed less gorging, removing less than half the amount of food compared to controls during the first 36 min of food access compared to controls (Fig. 3c); the amount of food withdrawn thereafter was similar to WT.

Restricted feeding was associated with reduced activity in the dark period and increased in activity in the lights-on period (Fig. 3d,e). Both responses were attenuated in *Mc3r*^−/−^ mice (Fig. 3e). The reduction in EE associated with RF (Fig. 3f) was primarily attributable to reduced REE; NREE appeared to be increased during RF that may suggest sparing of EE during periods of activity and a normal thermic effect of feeding (Fig. 3g). There was an effect of genotype when food access was restricted for 8 h, with the changes in REE and increase in NREE associated with RF being attenuated in *Mc3r*^−/−^ mice, despite normal food intake (compare Fig. 3b with 3f,g).

Restricting food access for 4 h requires mice to survive a 20-h period of fasting, and a decline in the RER between periods of food access indicated increased dependency on fatty acid reserves (Fig. 2c). The RER during the feeding period was similar between genotypes during the period of food access (1.025 ± 0.006 vs. 1.037 ± 0.009 for *Mc3r*^−/−^ and WT mice, respectively). However, a trend was observed for a lower RER in the 4-h period preceding food presentation in *Mc3r*^−/−^ mice (0.725 ± 0.002 vs. 0.732 ± 0.003; *t*_(14)_ = 1.994, *P* = 0.066). The duration of the increase in RER was also shortened in *Mc3r*^−/−^ mice, resulting in a trend for a smaller ‘area under the curve’ (4.067 ± 0.142 vs. 4.473 ± 0.133; *t*_(14)_ = 2.037, *P* = 0.061). The thermic effect of feeding, the increase in EE associated with food consumption, was also significantly lower in *Mc3r*^−/−^ mice during the 4 h of food access (1.7 ± 0.1 vs. 2.2 ± 0.2 kJ/h; *t*_(14)_ = 2.525, *P* < 0.05). Collectively, these data are all consistent with reduced food intake in *Mc3r*^−/−^ mice during RF relative to controls and an increased dependency on fatty acids released from adipose tissue for energy.

### Attenuated expression of anticipatory activity in Mc3r^−/−^ mice

**Figure 4:**
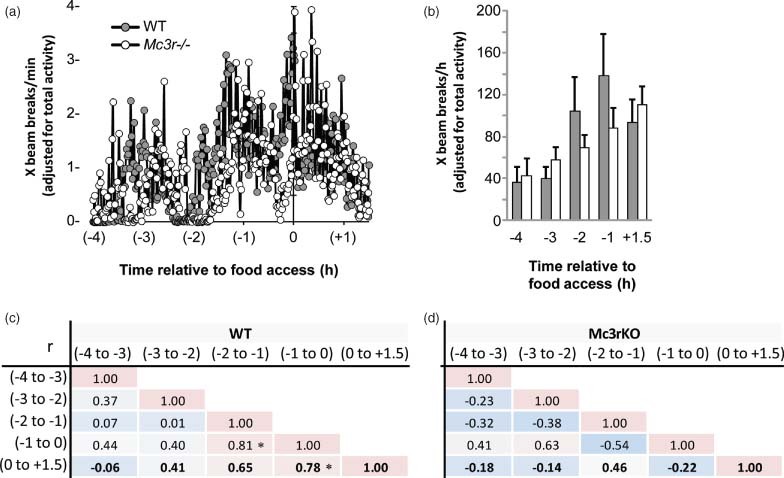
Attenuated expression of the food anticipatory response in *Mc3r*^−/−^mice (a) Locomotor activity data expressed as X-beam breaks per minute (adjusted for 24-h activity). WT mice display a gradual increase in the intensity of activity up to the time of food access. *Mc3r*^−/−^ mice display a delayed expression of anticipatory activity, while a peak in activity that coincides with food access in WT mice is not evident. (b) Expression of activity as 1-h bins showing the gradual increase in intensity of activity in WT that anticipate food presentation. (c) Correlation matrix displaying the relationship between activity in the 1-h intervals leading up to and the 90-min period following food access. WT mice display a gradual increase in consistency of activity leading up to food access (*P < 0.05). (d) In contrast, hourly activity of *Mc3r*^−/−^ mice displays more variability during the period leading up to food presentation.

As *Mc3r*^−/−^ mice displayed reduced locomotor activity (Fig. 3d), anticipatory activity was analyzed using data normalized to 24-h activity. Analysis of locomotor activity recorded in the 4-h preceding food presentation suggested reduced movement in *Mc3r*^−/−^ mice in the 2-h preceding food access (Fig. 4). Visual inspection of the raw activity data suggested that *Mc3r*^−/−^ mice were displaying reduced movement in the X-axis compared to WTs in the 2-h period prior to food access, and particularly in the 20–30 min period immediately preceding food access (Fig. 4a and data not shown). When anticipatory activity was assessed as 1-h bins (Fig. 4b–d), mice displayed a gradual increase in X-ambulatory movement in the time leading up to food access (Fig. 4b). Analysis using anova with repeated measures indicated a significant linear effect of time (*F*_1,14_ = 15.950, *P* < 0.01), with a trend for an interaction between time and genotype (*F*_1,14_ = 2.933, *P* = 0.10). However, excluding a WT mouse that displayed a decline in movement leading up to food presentation suggested an interaction between time and genotype (effect of time: *F*_1,13_ = 19.302, *P* < 0.01; time × genotype: *F*_1,13_ = 4.525, *P*≤ 0.05). Analysis of rearing activity (Z-movement) yielded a similar outcome (data not shown).

In the 2 h leading up to food access and in the period following food presentation, there were significant correlations in the level of activity of WT mice suggesting sustained expression of increased activity centered on food presentation (Fig. 4c). These correlations were not observed in *Mc3r*^−/−^ mice (Fig. 4d). Collectively, this analysis of ambulatory activity in the time leading up to food access is consistent with impaired adaptation of locomotor activity to RF, and an attenuated anticipation of food presentation in *Mc3r*^−/−^ mice.

### RF-associated DMH neuronal activity is attenuated in Mc3r^−/−^ mice

**Figure 5:**
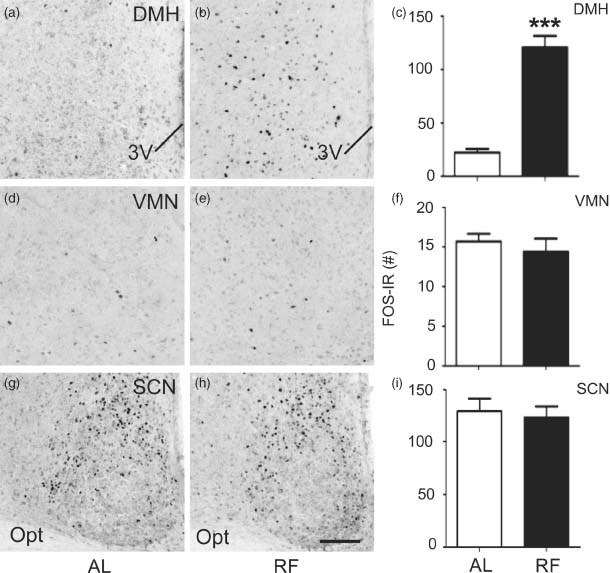
Increased DMH FOS-IR 90 min after food presentation in WT B6 mice subject to RF (a–c) RF is associated with increased neural activity, indicated by FOS-IR, within the DMH (representative photomicrographs at bregma level: −1.82 mm) compared to *ad libitum* (AL) feeding. In contrast, RF did not affect FOS-IR in the VMN (d–f; representative photomicrographs at bregma level: −1.58 mm) or SCN (g–i; representative photomicrographs at bregma level: −0.58 mm). Data in histograms (c, f and i) expressed as unilateral average ± SEM FOS-IR counts for each structure. ***P < 0.001 vs. *ad libitum*. The scale bar represents 100 µm.

We next assessed whether the regulation of neural activity in the hypothalamus during feeding is altered in *Mc3r*^−/−^ mice. First, we characterized the effect of RF on hypothalamic neural activity using WT B6 mice. FOS-IR was examined in the DMH (Fig. 5a–c), VMN (Fig. 5d–f) and SCN (Fig. 5g–i) in B6 mice fed *ad libitum* or subject to RF (*n* = 10/group). Brains were collected 90 min after food access in the RF group, and at the same time in controls fed *ad libitum*. There was no significant difference in FOS-IR between feeding conditions in the VMN or SCN. However, in the DMH, we detected a significant increase in FOS-IR in the RF group (Fig. 5a–c; DMH bregma level: −1.82 mm presented; *t*(7) = 6.083, *P* < 0.001). These data indicate that neurons in the DMH, but not SCN or VMN, are responsive to the RF paradigm utilized.

**Figure 6:**
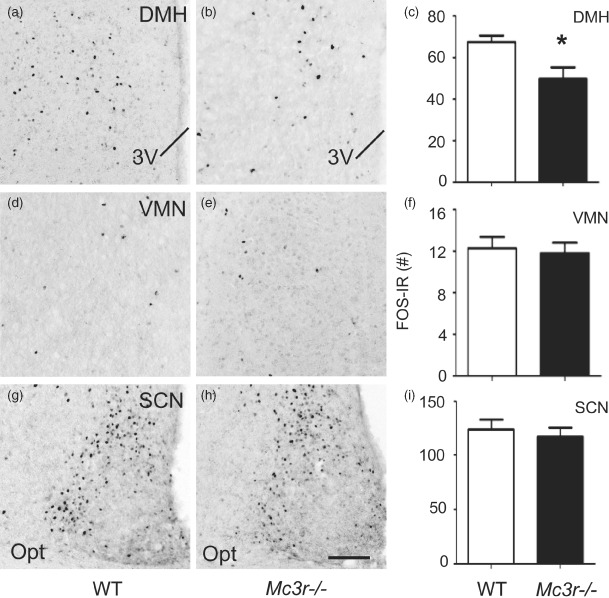
*Mc3r*^−/−^mice exhibit attenuated RF-associated neuronal activity in the DMH (a–c) Compared to WT controls (Fig. 5a–c), *Mc3r*^−/−^ mice subjected to RF exhibit an attenuation of neuronal activity, indicated by FOS-IR, specifically within the DMH at bregma level of −1.82 mm. No significant differences were detected in the VMN (d–f; representative photomicrographs at bregma level: −1.56 mm) or SCN (g–i; representative photomicrograph at bregma level: −0.58 mm). Data in histograms (c, f and i) expressed as unilateral average ± SEM FOS-IR counts. *P < 0.05 vs. WT. The scale bar represents 100 µm. Brain samples were collected 90 min after food access was granted, as shown in Fig. 1a.

To evaluate the potential role of MC3R in mediating this response, we assessed FOS-IR in the DMH (Fig. 6a–c), VMN (Fig. 6d–f) and SCN (Fig. 6g–i) of *Mc3r*^−/−^ and WT mice under RF conditions assessed in the CLAMS apparatus as above. *Mc3r*^−/−^ mice exhibited significantly blunted neuronal activity that was restricted specifically to the DMH in response to RF compared to WT controls (Fig. 6a–c; bregma level: −1.82 mm presented; *t*_(14)_ = 2.559, *P* < 0.05; see Fig. 5a–c). In contrast, FOS-IR expression was not significantly different between WT and *Mc3r*^−/−^ mice in the SCN or VMN. These data suggest that the impaired adaptive RF response displayed by *Mc3r*^−/−^ mice is associated with significantly reduced activity in the DMH.

### MC3R agonist increases DMH ERK activity

**Figure 7:**
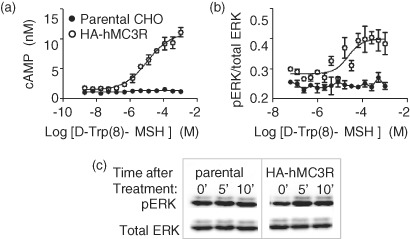
Dose-dependent increase in ERK phosphorylation in CHO cells expressing hMC3R treated withd-Trp(8)-*γ*MSH Dose-dependent effects of treatment were observed on cAMP accumulation (a) and pERK (b). (c) Assessment of pERK by western blot after treatment with 1 nm d-Trp(8)-*γ*MSH. Data shown are representatives of two stable CHO clones expressing an HA-tagged human MC3R.

To investigate the mechanism through which MC3R action influences DMH neuronal activity, we first examined the regulation of neural signaling by the MC3R agonist d-Trp(8)-*γ*MSH. We assessed MC3R signaling using stable CHO cell lines expressing hMC3R and observed a robust dose-dependent stimulation of both cAMP and pERK by d-Trp(8)-*γ*MSH (Fig. 7a,b). This response was also observed by western blots using a phospho-specific antibody (Fig. 7c).

**Figure 8:**
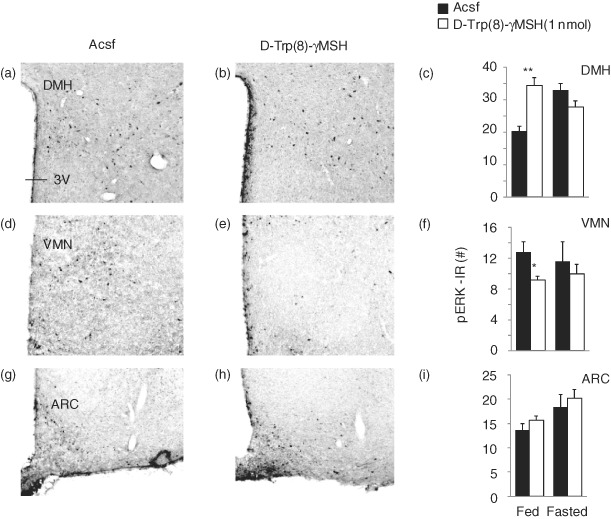
MC3R agonist increases the number of pERK-IR neurons in the DMH pERK-IR neurons in the DMH (a and b), VMN (d and e) and ARC (g and h) in control B6 mice treated with Acsf (a, d and g) or 1 nmol d-Trp(8)-*γ*MSH (b, e and h) fed *ad libitum* or after an overnight fast. The representative photomicrographs were taken using sections collected from mice treated in fed conditions. Treatment with d-Trp(8)-*γ*MSH significantly increased the number of pERK-IR neurons in the DMH in fed (representative photomicrograph at bregma level: −1.82 mm), but not fasting conditions (c). In the VMN, d-Trp(8)-*γ*MSH significantly reduced the number of pERK neurons (f) in fed, but not fasting conditions (representative photomicrograph at bregma level: −1.58 mm). There was no effect of treatment in the ARC (i) (representative photomicrograph at bregma level: −1.58 mm). *P < 0.05 vs. Acsf.**

To determine whether MC3R activation also influences pERK *in vivo*, we examined pERK staining in the ARC, VMN, DMH and PVN of male B6 mice in fed conditions or after an overnight fast state 15 min after intracerebroventricular injection of Acsf or the MC3R agonist d-Trp(8)-*γ*MSH (1 nmol) (Fig. 8; *n* = 5 per group). Significant effects of treatment were observed in the DMH and VMN, but not in the PVN or ARC (Fig. 8 and data not shown). In the DMH, there was an increase in the number of pERK-IR neurons in the d-Trp(8)-*γ*MSH treatment group in fed conditions, but not in fasted conditions (Fig. 8a–c; bregma level: −1.82 mm). Analysis using two-way anova indicated a significant effect of treatment (*F*_1,16_ = 5.211, *P* < 0.05), with a significant interaction between treatment and fed condition (*F*_1,16_ = 24.808, *P* < 0.01). Regulation of pERK was also observed in the VMN, with the number of pERK-IR neurons being reduced by d-Trp(8)-*γ*MSH treatment in fed conditions (8D-F; bregma level: −1.58 mm). Analysis using two-way anova indicated a significant effect of treatment (*F*_1,15_ = 8.157, *P* < 0.05). In the ARC, regulation of pERK was observed by fed condition only (*F*_1,16_ = 8.320, *P* < 0.05), with no effect of treatment (Fig. 8g–i and data not shown). In the PVN, a modest (10%) effect of treatment was observed (*F*_1,16_ = 5.571, *P* < 0.05), with no effect of feeding condition (data not shown). Collectively, these data suggest that activation of MC3Rs can influence pERK and that *in vivo* the administration of d-Trp(8)-*γ*MSH increases pERK in the DMH in conditions of nutritional sufficiency.

## Discussion

The functions of MC3Rs expressed in the hypothalamus and limbic regions of the brain remain enigmatic. Our recent investigation suggests a role for MC3Rs in the expression of rhythms anticipating nutrient intake. *Mc3r*^−/−^ mice display circadian-associated alterations including reduced expression of rhythms anticipating food intake and increased dependency on photic cues to maintain rhythms (Begriche *et al.* 2011a,b; Sutton *et al.* 2008, 2010). We also reported that the *Mc3r*^−/−^ circadian phenotype is associated with arrhythmic expression of clock genes in the forebrain and liver, indicating that MC3R regulate inputs that synchronize clock activity with signals of food intake (Begriche *et al.* 2011b; Sutton *et al.* 2008, 2010). Here, we report that *Mc3r*^−/−^ mice adapted poorly to a RF schedule, displaying significant alterations in food intake, locomotor activity, EE and weight loss relative to WT controls. The results presented here are also significant for suggesting that MC3R signaling may influence the activity of neurons residing in the DMH that some studies suggest are involved in the expression of feeding-related rhythms in behavior (Chou *et al.* 2003; Gooley *et al.* 2006; Mieda *et al.* 2004, 2006). These observations provide further evidence indicating that one of the homeostatic functions of MC3R is synchronizing peaks in vigilance with nutrient availability.

The hypothesis that a clock-like system entrains rhythms with calorie intake independently of the light-entrainable oscillator residing in the SCN dates back to the 1970s (Stephan 2002). The earliest efforts involved investigating how mechanical lesions altered rhythms indicated that food entrainment might involve neurons in the VMN and are independent of SCN output (Choi *et al.* 1998; Krieger 1980; Krieger *et al.* 1977; Mistlberger & Rechtschaffen 1984; Stephan *et al.* 1979). Studies using excitotoxic lesions performed with increased precision further defined specific regions outside that SCN have functions required for the expression of rhythms related to food presentation (Chou *et al.* 2003; Gooley *et al.* 2006). The results reported by Gooley *et al.* (2006) suggested that a functional DMH is required for acquisition and full expression of rhythms anticipating food presentation, while the VMN might have an inhibitory role. Another laboratory reported entrainment of oscillations in *Per2* expression, an indicator of clock activity, in the DMH to daily food presentation that persisted in the absence of food intake (Mieda *et al.* 2006). We observed that MC3R may be involved in transmitting feeding-related signals to the DMH. Specifically, we observed that expression of FOS-IR activity in the DMH following food intake is attenuated in *Mc3r*^−/−^ mice, indicating a role for this receptor in mediating signals of food intake into this region. These data are consistent with previous reports indicating increased FOS-IR in the DMH and LHA after the ingestion of food in mice (Hsu *et al.* 2010) and rats (Johnstone *et al.* 2006; Singru *et al.* 2007), and involves activation of melanocortin neurons expressing the precursor for the endogenous melanocortin receptor agonists (Johnstone *et al.* 2006; Singru *et al.* 2007).

Complementing the *Mc3r*^−/−^ phenotype, we also report that central administration of a MC3R agonist increases neural activity in the DMH in fed conditions. Specifically, central administration of d-Trp(8)-*γ*MSH, an analog of *γ*−MSH (Grieco *et al.* 2000), rapidly increased the number of pERK-IR neurons in the DMH. The mechanisms explaining the observed response to d-Trp(8)-*γ*MSH in fed but not fasted conditions are not clear. However, one possible explanation is inhibition of agonist activity resulting from increased expression and release of agouti-related peptide, a competitive MC3R antagonist, that is observed with fasting (Hahn *et al.* 1998; Mizuno & Mobbs 1999; Wilson *et al.* 1999). This result should also be interpreted with caution, as d-Trp(8)-*γ*MSH also exhibits agonist activity at MC4R (Grieco *et al.* 2000). However, previous analysis of pERK in rats treated with melanotan-II suggested regulation by MC4R restricted to oxytocin-expressing neurons in the PVN (Daniels *et al.* 2003). We also observed d-Trp(8)-*γ*MSH stimulation of pERK in CHO cells. These data are consistent with a report indicating that MC3Rs regulate ERK activity in HEK 293 cells (Chai *et al.* 2007), but are not consistent with a report using COS-1 cells expressing MC3R (Daniels *et al.* 2003).

Another interesting observation is the divergence in the effect of d-Trp(8)-*γ*MSH treatment on the number of pERK-IR neurons in the DMH and VMN. While d-Trp(8)-*γ*MSH treatment increased the number of pERK-IR neurons in the DMH, it had the opposite effect in the VMN. The VMN displays dense *Mc3r* mRNA expression (Roselli-Rehfuss *et al.* 1993). The response of glutamatergic VMN neurons to several melanocortin receptor agonists (including d-Trp(8)-*γ*MSH) has also been investigated using whole-cell voltage clamp and current-clamp electrophysiological recordings in hypothalamic slices (Fu & van den Pol 2008). Generally, all melanocortin receptor agonists were found to have an inhibitory effect on the activity of VMN glutamatergic neurons. These effects were indirect, involving modulation of GABA-ergic synaptic inputs. Our observation of a suppression of pERK activity in the VMN by d-Trp(8)-*γ*MSH is thus in general agreement with an inhibitory impact on the activity of VMN neurons, although we did not characterize the neurons displaying pERK activity. At this time, it is also not possible to ascertain whether the impact of d-Trp(8)-*γ*MSH on pERK is direct or indirect; although the latter appears to be more likely based on the electrophysiological data mentioned above and our analysis of MC3R signaling *in vitro* suggesting that activation should stimulate pERK. With respect to the functions of MC3Rs in the VMN, we have also examined the specific role of these receptors in energy homeostasis (Begriche *et al.* 2011a). Our analysis suggests that selectively rescuing VMN MC3R signaling is not sufficient to regulate the expression of FAA as assessed using wheel running activity, and that these receptors may have functions related to metabolic homeostasis.

It is however important to note the limitations of the current studies and that expression of FAA has been observed following DMH lesions by some groups (Landry *et al.* 2006, 2007). FOS-IR was measured after food presentation; we therefore cannot draw definitive conclusions about the role of DMH neurons in the expression of anticipatory activity based on the current methodological approach. Determining whether MC3R signaling in these neurons is required specifically for the expression of food anticipatory rhythms will require further experimentation; for example, we can use a recently developed inducible MC3R expression model to determine whether MC3R expression in the DMH alone is sufficient to rescue this behavior (Begriche *et al.* 2011a). Our data suggests that the presence of neurons in the DMH of mice are sensitive to calorie intake, and that full expression of the increased FOS-IR in the DMH during feeding involves MC3R signaling.

In conclusion, these studies suggest a hypothalamic MC3R-dependent signaling pathway that may be involved in regulating the homeostatic responses to signals of nutrient intake, and support a physiological consequence of loss of MC3R signaling during RF. These studies have begun to assess signaling mechanisms that could mediate this response via MC3R activation in the DMH. The possibility that MC3Rs expressed in the DMH link signals of food intake with expression of anticipatory rhythms merits further study.
